# Serological differentiation of West Nile, Usutu, and tick-borne encephalitis virus antibodies in birds and horses using mutant E protein ELISAs

**DOI:** 10.1038/s41598-025-14448-4

**Published:** 2025-08-06

**Authors:** Anne Schwarzer, Ute Ziegler, Jasmin Fertey, Markus Kreuz, Thomas W. Vahlenkamp, Martin H. Groschup, Sebastian Ulbert

**Affiliations:** 1 Federal Research Institute for Animal Health, Institute of Novel and Emerging Infectious Diseases, Friedrich-Loeffler Institut, Südufer 10, 17493 Greifswald-Insel Riems, Germany; 2 Fraunhofer Institute for Cell Therapy and Immunology, Department of Infection Research and Diagnostics, Perlickstraße 1, 04103 Leipzig, Germany; 3 Fraunhofer Institute for Cell Therapy and Immunology, Department of Medical Bioinformatics,, Perlickstraße 1, 04103 Leipzig, Germany; 4 Institute of Virology, Faculty of Veterinary Medicine, Leipzig University, An den Tierkliniken 29, 04103 Leipzig, Germany

**Keywords:** West nile virus, Usutu virus, Tick-borne encephalitis virus, Cross-reactivity, Envelope protein, ELISA, Viral infection, Applied immunology

## Abstract

West Nile virus (WNV), Usutu virus (USUV) and tick-borne encephalitis virus (TBEV) are worldwide endemic zoonotic orthoflaviviruses, often co-circulating in the same areas. Serological studies in animals, mostly birds and horses, are important means to monitor the spread of these viruses and the infection risks for humans. However, cross-reactive antibodies to these structurally similar flaviviruses frequently impact serological differentiation in enzyme-linked immunosorbent assays (ELISAs), hence time-consuming virus neutralization tests (VNTs) have to be employed in laboratories with high biosafety level. This study presents ELISAs using recombinant flavivirus E proteins with point mutations in the conserved fusion loop domain (Equad proteins) for differentiating IgY or IgG antibodies against WNV, USUV or TBEV in ducks, geese, chickens and horses. Panels of 169 duck and goose sera, 101 chicken sera and 136 horse sera were tested in Equad ELISAs, which resulted in high sensitivity and specificity, further improved by a pre-absorption step for the differentiation of WNV and USUV antibodies. Equad ELISAs for poultry and horse sera enable the reliable differentiation of WNV, USUV and TBEV specific antibodies without the need for VNTs, which has important implications for conducting seroprevalence studies as well as for veterinary routine diagnosis.

**Supplementary Information:**

The online version contains supplementary material available at 10.1038/s41598-025-14448-4.

## Introduction

Zoonotic, arthropod-borne flaviviruses such as West Nile virus (WNV, *Orthoflavivirus nilense*), Usutu virus (USUV, *Orthoflavivirus usutense)* and tick-borne encephalitis virus (TBEV, *Orthoflavivirus encephalitidis* (International Committee on Taxonomy of Viruses 2024)) belong to the *Flaviviridae* family and are known to cause infections ranging from mild symptoms to fatal outcomes in animals including mammals^1^.

WNV, USUV and TBEV are endemic in many countries worldwide, including Europe. Changing climate conditions might contribute to a further spread of vector-borne flaviviruses in the future^2,3^. In Germany, WNV is endemic in the central-eastern part. Epidemiological studies point to a notable spread northward and towards the southwest in 2024^4–6^. WNV was introduced to Germany from the Czech Republic and Austria via migratory birds and was first isolated in dead birds in 2018 in Germany^7^. To date, up to nine WNV lineages are known worldwide, with lineages 1 and 2 capable of causing clinical infections in humans and animals^8^. USUV has already spread throughout the entire country and was first isolated in Germany in 2010 from mosquitoes and later from dead birds in 2011^9^. At that time the distribution was limited to the upper Rhine valley and neighboring regions in southwestern Germany which changed during a USUV outbreak in 2018 resulting in high mortality among blackbirds and frequent deaths of owls in aviaries^10^. Eight USUV Lineages are known worldwide^11^. TBEV consists of three main subtypes that circulate in Europe^12^, including Germany^13^. Vaccines are available to protect humans from TBEV and horses from WNV.

The transmission of WNV, USUV and TBEV depends on the seasonal activity of their arthropod vectors. The primary vectors for WNV and USUV are mosquitoes of the *Culex pipiens* complex^14,15^. WNV and USUV mainly infect birds, which act as reservoir hosts and amplifiers of the viruses^11,16,17^. Mammals such as horses or humans can also be infected by mosquitoes^18^. They, however, represent dead-end hosts^16,19,20^. Ticks, mostly belonging to the *Ixodes ricinus* complex, predominantly transmit TBEV to small mammals^21^. The zoonotic potential of WNV, USUV and TBEV highlights the need for rapid and reliable screening methods for surveillance. As viral RNA is detectable only for a short time upon flavivirus infection, due to the short duration of viremia, antibody measurements are mostly used in this context.

Specific serological diagnosis of flavivirus infections is challenging due to the antigenic similarity among closely related flaviviruses, which results in highly cross-reactive antibody responses. Different approaches are being used to increase specificity of diagnostics^22–24^.

Currently, pan-flavivirus enzyme-linked immunosorbent assays (ELISAs) and immunofluorescence assays (IFAs) are commonly employed in flavivirus diagnostics because of their high sensitivity. However, these assays often exhibit low specificity due to cross-reactive binding among antigenically related flaviviruses, especially within the same serocomplex^25^ as is the case with WNV and USUV^16,26^. As a consequence, virus neutralization tests (VNTs) are considered the gold-standard for specificity, but they have several disadvantages: demand of time (several days), high labor and material costs and the need for a biosafety level 3 (BSL3) laboratory for WNV and TBEV. In order to overcome these disadvantages, it is highly relevant to reach an improvement in the specificity of ELISAs, which are preferred for screening due to their time-efficiency and suitability for high-throughput applications^27^, with the aim of reducing or eliminating the need for VNTs confirmation in the future.

The envelope (E) protein of flaviviruses is highly immunogenic during infection^28^ and therefore commonly used for antibody detection. It consists of three domains: EDI, EDII and EDIII^29^. A highly conserved region within EDII, known as the fusion loop (FL), is a primary target for cross-reactive antibodies^30^, contributing to the low specificity of serological tests relying on the E protein. Previous studies have demonstrated that mutations in the FL can reduce cross-reactive binding of antibodies^31–35^. These recombinant mutant envelope proteins (termed Equad proteins) have been used for dengue virus and Zika virus diagnostics^36^ and for differentiation of WNV- and USUV-induced antibodies in human sera^35^ as well as in a pilot study aimed to distinguish TBEV and WNV IgG antibodies in horse sera^37^. However, Equad proteins have not yet been tested for birds, which can play an important role as a sentinel for WNV surveillance.

In order to determine whether Equad proteins can effectively and reliably differentiate flavivirus antibodies in avian and equine sera, corresponding ELISAs were established for WNV, USUV and TBEV.

## Methods

### Antigens

Equad proteins (ectodomains of the E protein containing four point mutations near the FL domain) for WNV, USUV and TBEV were produced in a *Drosophila* S2 cell expression system (Thermo Fisher, Waltham, USA) and purified as previously described by Rockstroh et al.^34^ and Berneck et al.^35^.

### Avian serum samples

Avian serum samples (*n* = 271) from chickens (*Gallus sp.*), ducks (*Anatidae sp.*) and geese (*Anserinae sp.*) from experimental studies as well as field sera from the National Reference Laboratory (NRL) were included in this study. Flavivirus IgY-positive poultry samples (*n* = 127) were tested positive for either WNV, TBEV or USUV. A comparable set of flavivirus IgY-negative sera from chickens, ducks and geese (*n* = 144) was also included. A detailed list of the origin of each serum sample can be found in Supplementary Tables S1 – S7. All serum samples were characterized with regard to their flavivirus antibody titers using a commercially available competitive ELISA (ID Screen^®^ WN competition ELISA, IDVet, Grabels, France) followed by virus neutralization tests (VNT) as described by Seidowski et al.^38^ using the mammalian cell lines Vero B4, Vero 76 and PK15, which were obtained from the collection of Cell Lines in Veterinary Medicine (Friedrich-Loeffler-Institut (FLI), Germany). Duck and goose sera were combined into a single group because preliminary tests during protocol establishment indicated that both require the same procedures in the different ELISAs. Detailed information on the flavivirus antibody status and the amount of tested avian sera is provided in Tables 1 and 2 for Equad ELISAs and Equad pre-absorption ELISAs, respectively.

### Equine serum samples

Equine field serum samples from the NRL as well as sera from routine diagnostics in veterinary clinics are part of this study. A detailed list of the origin of each serum sample can be found in Supplementary Tables S8 – S11. Information regarding the antibody status of the equine serum samples (*n* = 136) and the number of samples tested is presented in Tables 1 and 2. Samples were classified as either positive for WNV, USUV or TBEV or flavivirus antibody negative. The samples were examined to determine their Flavivirus IgG antibody titers using a commercially available competition ELISA (ID Screen^®^ WN competition ELISA, IDVet, Grabels, France) followed by VNTs on sera that yielded positive ELISA results.

### Determination of antibody status of the serum samples

Pre-characterization of the sera utilized for the Equad and Equad pre-absorption ELISAs was conducted employing commercially available ELISAs and VNTs. Sera were characterized with regard to their flavivirus antibody titers using the ID Screen^®^ Flavivirus competition ELISA (formerly named West Nile competition ELISA at the time of testing, IDVet, Grabels, France). Sera that yielded positive results were further tested in WNV, USUV and TBEV virus neutralization tests for which the mammalian cell lines Vero B4, Vero 76 and PK15 were used. The corresponding VNT titers to the positive sera of the panel are provided in the Supplementary Tables S1-S11. Positive sera from naturally infected birds were tested against WNV and USUV in the VNT to ensure reliable differentiation. The experimentally infected geese and chickens were purchased as day-old chicks or SPF-free hatching eggs^39–41^. The TBEV positive duck sera originated from an experimental infection where animals were purchased at four weeks of age and tested negative for TBEV in the VNT^42^, which theoretically leaves the possibility of a heterologous flavivirus infection. Positive horse sera were tested against WNV, USUV and TBEV in the VNT. However, a few isolated sera constitute exceptions to this pattern. These sera originate from regions and years in which the presence of antibodies against WNV, USUV, or TBEV could be ruled out with certainty, as these viruses were not present at the time and in the area in question. These details are provided for each serum in the Supplementary Tables S8 - S11. The VNT titer for the virus for which the sample was tested positive was always at least four times higher than VNT titers of the other flaviviruses tested, in accordance to standard procedures^43^. This does not exclude the slight possibility that animals do have antibody titers against a heterologous flavivirus that are less than four times lower than those against the flavivirus under investigation.

### ELISA setup

Different indirect ELISA assays using Equad proteins as antigens were established for the testing of either chicken, duck and goose or horse sera for WNV, USUV or TBEV IgY/IgG antibodies. To define optimal test conditions, different serum sample dilutions (1:10, 1:50, 1:100) and several secondary antibodies at varying dilutions (1:10,000, 1:20,000, 1:50,000) were compared for avian sera. Furthermore, incubation at room temperature or at 37 °C was compared.

After optimization for chicken serum samples either 160 ng of WNV Equad protein or 200 ng of TBEV Equad protein per well in 100 µl ELISA coating buffer (15 mM Na_2_CO_3_, 35 mM NaHCO_3_, pH 9.6) were applied onto Nunc 96 well polysorb plates (Thermo Scientific) at 4°C overnight. The following day, the plates were washed three times with 350 µl PBS-0.05% Tween20 per well and then blocked with 200 µl of 5% skimmed milk (blocking grade) in PBS for two hours at room temperature. After another washing step, 100 µl of 1:100 diluted chicken sera in skimmed milk in PBS were added to each well and incubated for one hour at room temperature. Following another washing step, 100 µl of the HRP-conjugated secondary antibody (goat anti-bird IgG antibody, Bethyl Laboratories) was added to each well at a dilution of 1:50,000 and the plates were incubated for one hour at room temperature. A final washing step was performed, followed by the addition of 100 µl TMB substrate (BioLegend) per well for 30 min at room temperature in the dark. The reaction was then stopped with 50 µl 1M H_2_SO_4_ per well. The signals were read out at 450 nm with background reduction at 520 nm using a micro plate reader (TECAN infinite M200 or TECAN infinite F200 pro).

For duck and goose serum samples, the chicken serum protocol was adapted to a serum dilution of 1:50 and a secondary antibody dilution of 1:10,000. In addition, duck and goose sera were tested using either WNV Equad protein (160 ng per well), USUV Equad protein (200 ng per well) or TBEV Equad protein (200 ng per well).

Equine serum samples were tested in WNV Equad, TBEV Equad and USUV Equad IgG ELISAs. The protocol for equine samples was identical to the protocol described for chicken sera, except for the additional testing on USUV Equad protein (200 ng per well) and using a HRP-conjugated rabbit anti-horse IgG H&L (abcam) as secondary antibody at a dilution of 1:50,000.

WNV and USUV Equad pre-absorption ELISAs were established to further reduce cross-reactive binding of IgG and IgY antibodies to WNV and USUV Equad proteins in horse, duck and goose sera. The diluted sera were pre-incubated with the heterologous antigen for one hour at room temperature before being added to the wells. For this pre-absorption step, 20 µg per mL of USUV Equad protein in the WNV Equad pre-absorption ELISA or 16 µg per mL of WNV Equad protein in the USUV Equad pre-absorption ELISA were used. The other steps of the ELISA protocol were identical to those described above for duck, goose and horse sera. The amount of heterologous antigen in the pre-absorption step was determined according to Berneck et al. 2022^35^.

### Statistical analysis

The mean value of all measurements for each serum was included in the statistical analysis. Statistical analysis was performed using GraphPad Prism 6 (GraphPad Software, Inc, La Jolla, CA, USA). The results from the different ELISA setups were compared among sample groups based on their flavivirus IgG/ IgY antibody status using boxplots. For each ELISA, receiver operating characteristics (ROC) analyses were performed and the area under the curve (AUC) with a 95% confidence interval was calculated. The cut-off values for each ELISA setup were calculated using Youden’s index, which equally weights false positive and false negative values following ROC analysis. Samples were considered positive if their OD values exceeded the calculated cut-off value and sensitivity and specificity were determined.

### Ethics declarations

All methods are reported in accordance with ARRIVE guidelines. All methods were carried out in accordance with relevant guidelines and regulations. Duck, goose and chicken sera were used according to the ethical approvals by the administration of the federal state of Mecklenburg-Western Pomerania, Germany (LALLF reference numbers 7221.3-2-042/17, 7221.3-2-003/23, 7221.3-1-075/16, 7221.3-1.1-018/18 and 7221.3-1-031/20). All experimental protocols were approved by the administration of the federal state of Mecklenburg-Western Pomerania. Blood samples from horses were collected during routine diagnostic testing by veterinarians for which, according to German law, ethical approval was not required.

## Results

### Development of serum panels characterized by competition ELISA and VNTs

Serum panels for the Equad ELISAs and Equad pre-absorption ELISAs were categorized into three groups: duck and goose sera were combined into a single group, while chicken sera constituted the second group and horse sera comprised the third group. All sera were tested using the commercially available Flavivirus competition ELISA and positive sera were further characterized by WNV, USUV and TBEV VNTs. Tables 1 and 2 provide an overview of the exact composition of the serum panel. A total of 170 duck and goose serum samples were analyzed, of which 108 were found to be flavivirus antibody negative (one of these sera was tested exclusively in the pre-absorption ELISA), 31 were WNV IgY antibody positive, eight were USUV IgY antibody positive, and 23 were TBEV IgY antibody positive. The panel included 101 chicken serum samples, of which 36 were found to be flavivirus IgY negative, 19 were WNV IgY antibody positive, and 46 were TBEV IgY antibody positive. Furthermore, 136 horse sera were included in the panel. Of these, 41 were found to be flavivirus IgG negative, 49 were WNV IgG antibody positive, nine were USUV IgG antibody positive and 37 were TBEV IgG antibody positive. The corresponding VNT titers to the positive sera of the panel are provided in the Supplementary Tables S1-S11.

**Table 1:** Samples in Equad ELISAs and their flavivirus antibody status.

Duck and goose sera:		Chicken sera:		Horse sera:	
Flavivirus IgY antibodies detected via commercial ELISA and VNTs	Number of sera	Flavivirus IgY antibodies detected via commercial ELISA and VNTs	Number of sera	Flavivirus IgG antibodies detected via commercial ELISA and VNTs	Number of sera
Negative	107	Negative	36	Negative	41
Positive (WNV/ USUV/ TBEV)	62 (31/ 8/ 23)	Positive (WNV/ USUV/ TBEV)	65 (19/ 0/ 46)	Positive (WNV/ USUV/ TBEV)	95 (49/ 9/ 37)
**Total: 169 samples**		**Total: 101 samples**		**Total: 136 samples**	

**Table 2:** Samples in Equad pre-absorption ELISAs and their flavivirus antibody status.

Duck and goose sera:		Horse sera:	
Flavivirus IgY antibodies detected via commercial ELISA and VNTs	Number of samples	Flavivirus IgG antibodies detected via commercial ELISA and VNTs	Number of samples
Negative	23	Negative	23
Positive (WNV/ USUV)	39 (31/ 8)	Positive (WNV/ USUV)	58 (49/ 9)
**Total: 62 samples**		**Total: 81 samples**	

### Equad ELISAs and Equad pre-absorption ELISAs

Equad proteins for WNV, USUV and TBEV were utilized as antigens in ELISAs to detect IgY or IgG antibodies against either WNV, USUV or TBEV. Consequently, sera from the panel were evaluated using in total twelve Equad and Equad pre-absorption ELISA protocols. The pre-absorption step consists of the incubation of the test serum with the heterologous antigen before adding the sample to the ELISA-plate, where the homologous antigen is coated. Antibody measurements using ELISAs were carried out in duplicates in at least two independent experiments. The results are shown according to the three groups in the serum panel.

#### Duck and goose sera

**Fig. 1:**
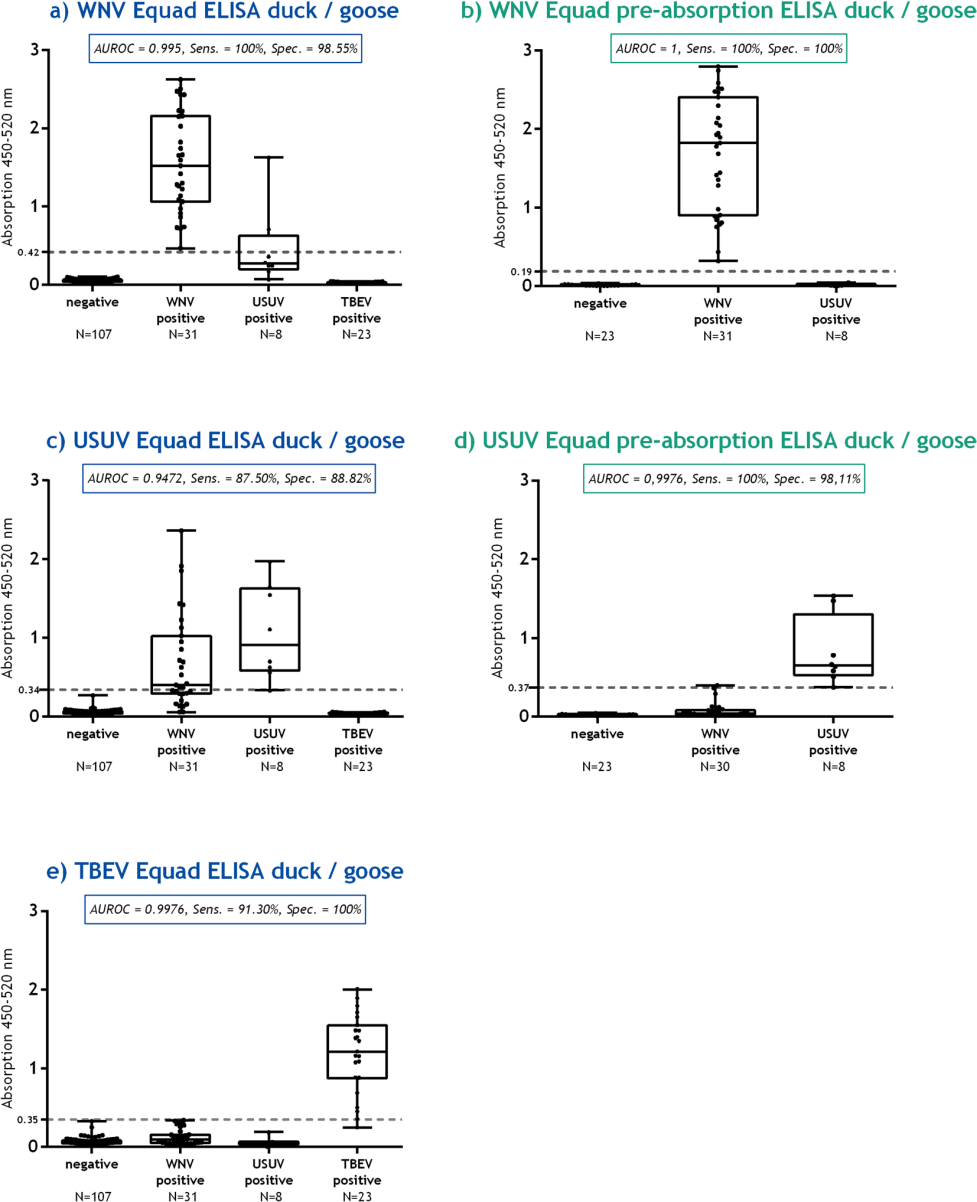
Equad and Equad pre-absorption ELISAs for the differentiation of flavivirus IgY antibodies in duck and goose sera. The sera are categorized according to their antibody status on the x-axis, as verified by virus neutralization tests. The graphs display the area under the curve in receiver-operating characteristic (AUROC) with sensitivity (Sens.) and specificity (Spec.). The calculated cut-off value is indicated by a dashed gray line on the y-axis. (**a)**: WNV Equad ELISA duck and goose sera, 95%CI 0.9853 to 1.005. **(b)**: WNV Equad pre-absorption ELISA duck and goose sera, 95%CI 1.000 to 1.000. (**c)**: USUV Equad ELISA duck and goose sera, 95%CI 0.9103 to 0.9841. (**d)**: USUV Equad pre-absorption ELISA duck and goose sera, 95%CI 0.9900 to 1.005.(**e)**: TBEV Equad ELISA duck and goose sera, 95%CI 0.9925 to 1.003.

A total of 170 duck and goose sera was analyzed (Tables 1 and 2) in five different ELISA setups (Fig. 1a - e). The corresponding raw data, as well as detailed information on the sera and associated VNT titers, are provided in Supplementary Tables S1 - S4. For the WNV Equad ELISA for duck and goose sera a sensitivity of 100% was achieved, the specificity was 98.55% at a cut-off value of 0.42 (Fig. 1a) with an area under the curve in ROC analysis (AUROC) of 0.995 (S1 Fig). For the USUV Equad ELISA, sensitivity and specificity were both 87.50% and 88.82%, respectively, at a cut-off value of 0.34 (Fig. 1c) with an AUROC of 0.9472 (S3 Fig). In order to minimize cross-reactive binding in subsequent analyses, the WNV and USUV IgY antibody positive sera were analyzed in WNV Equad pre-absorption and in USUV Equad pre-absorption ELISAs, so the sera were pre-incubated with USUV or WNV Equad protein, respectively. This additional step in the WNV Equad pre-absorption ELISA increased the sensitivity and specificity to 100% at a cut-off value of 0.19 (Fig. 1b), leading to an optimal AUROC of 1 (S2 Fig). In the USUV Equad pre-absorption ELISA, one WNV-positive serum was excluded due to insufficient sample volume, leaving 30 WNV-positive samples for statistical analysis. For the USUV Equad pre-absorption ELISA, the sensitivity also remained 100% and the specificity was increased to 98.11% at a cut-off value of 0.37 (Fig. 1d) with an AUROC of 0.9976 (S4 Fig). Among the eight USUV positive samples, two sera showed a false positive result in the WNV Equad ELISA (25.00%). However, in the WNV Equad pre-absorption ELISA, none of the eight sera were detected false positive. In addition, a TBEV Equad ELISA was performed (Fig. 1e). The ROC analysis showed a sensitivity of 91.30% and a specificity of 100% at a cut-off value of 0.35 with an AUROC of 0.9976 (S5 Fig). Four of the TBEV positive duck sera had VNT titers ranging from 1:15 to 1:40 and showed OD values < 0.5 in the TBEV-Equad-ELISA, which was comparable to the OD values of some WNV positive samples. Although these sera had low VNT titers, two of the four exceeded the cut-off value and could therefore be classified as TBEV positive. Given the results in specificity and the fact that TBEV belongs to a different serocomplex than WNV and USUV, performing a pre-absorption ELISA for TBEV was not necessary.

#### Chicken sera

**Fig. 2:**
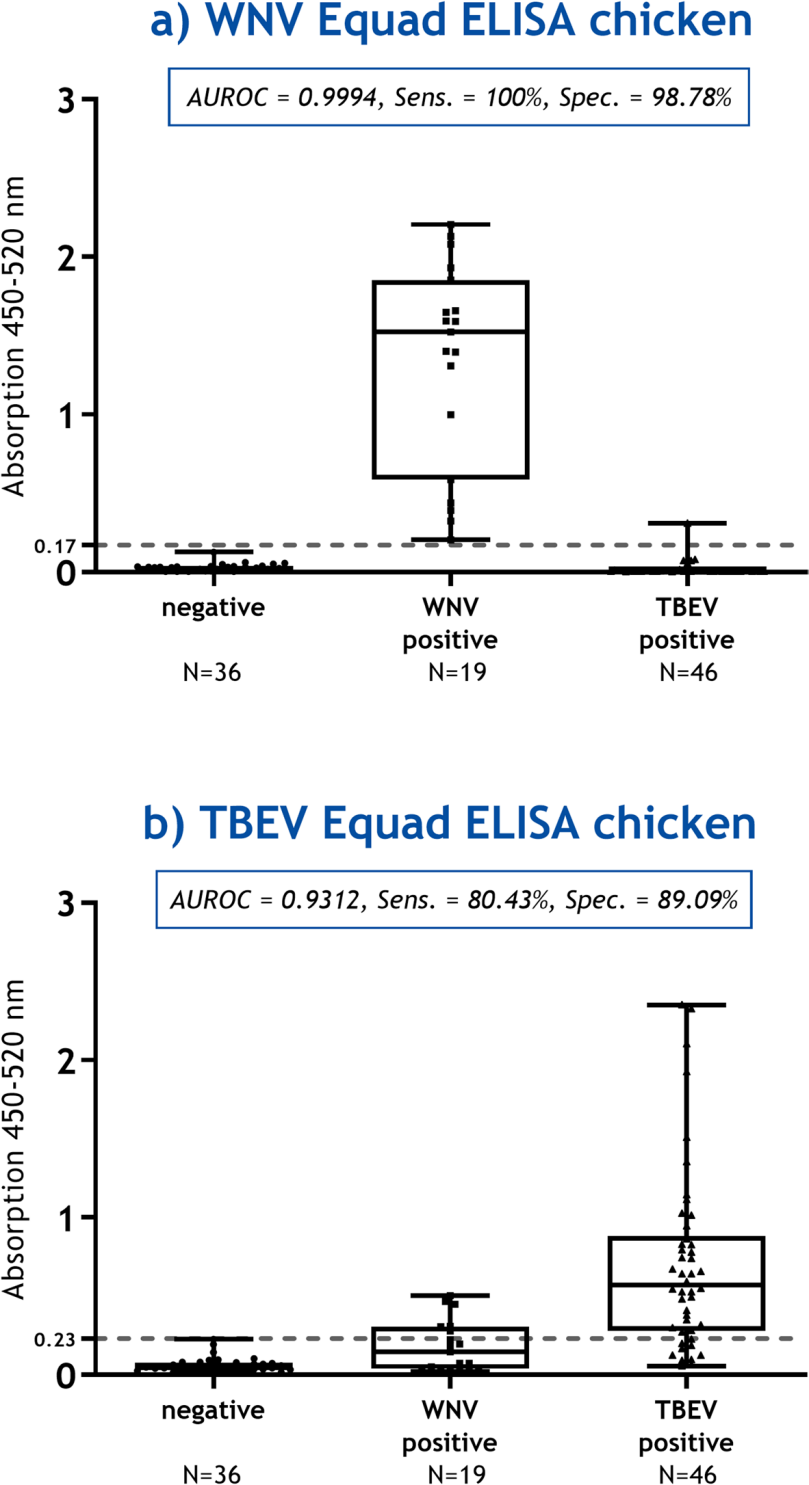
Equad ELISAs for the differentiation of flavivirus IgY antibodies in chicken sera. The sera are categorized according to their antibody status on the x-axis, as verified by virus neutralization tests. The graphs display the area under the curve in receiver-operating characteristic (AUROC) with sensitivity (Sens.) and specificity (Spec.). The calculated cut-off value is indicated by a dashed gray line on the y-axis. **(a)**: WNV Equad ELISA chicken sera, 95%CI 0.9972 to 1.001. **(b)**: TBEV Equad ELISA chicken sera, 95%CI 0.8861 to 0.9764.

A total of 101 chicken serum samples (Table 1) were analyzed using a WNV Equad ELISA (Fig. 2a) and a TBEV Equad ELISA (Fig. 2b). The corresponding raw data can be found in Supplementary Tables S5 – S7. Due to a lack of USUV positive chicken sera, USUV antibodies were not analyzed. Slight cross-reactive binding between WNV and TBEV IgY antibodies was observed resulting in a cut-off value of 0.17 with a sensitivity of 100% and a specificity of 98.78% (S6 Fig) in the WNV Equad ELISA. In the TBEV Equad ELISA, a sensitivity of 80.43% and specificity of 89.09% were determined with an AUC in ROC analysis of 0.9312 at a cut-off value of 0.23 (S7 Fig). Due to the currently limited relevance of TBEV for chicken we have not used the pre-absorption setup to further improve specificity.

#### Equine sera

**Fig. 3:**
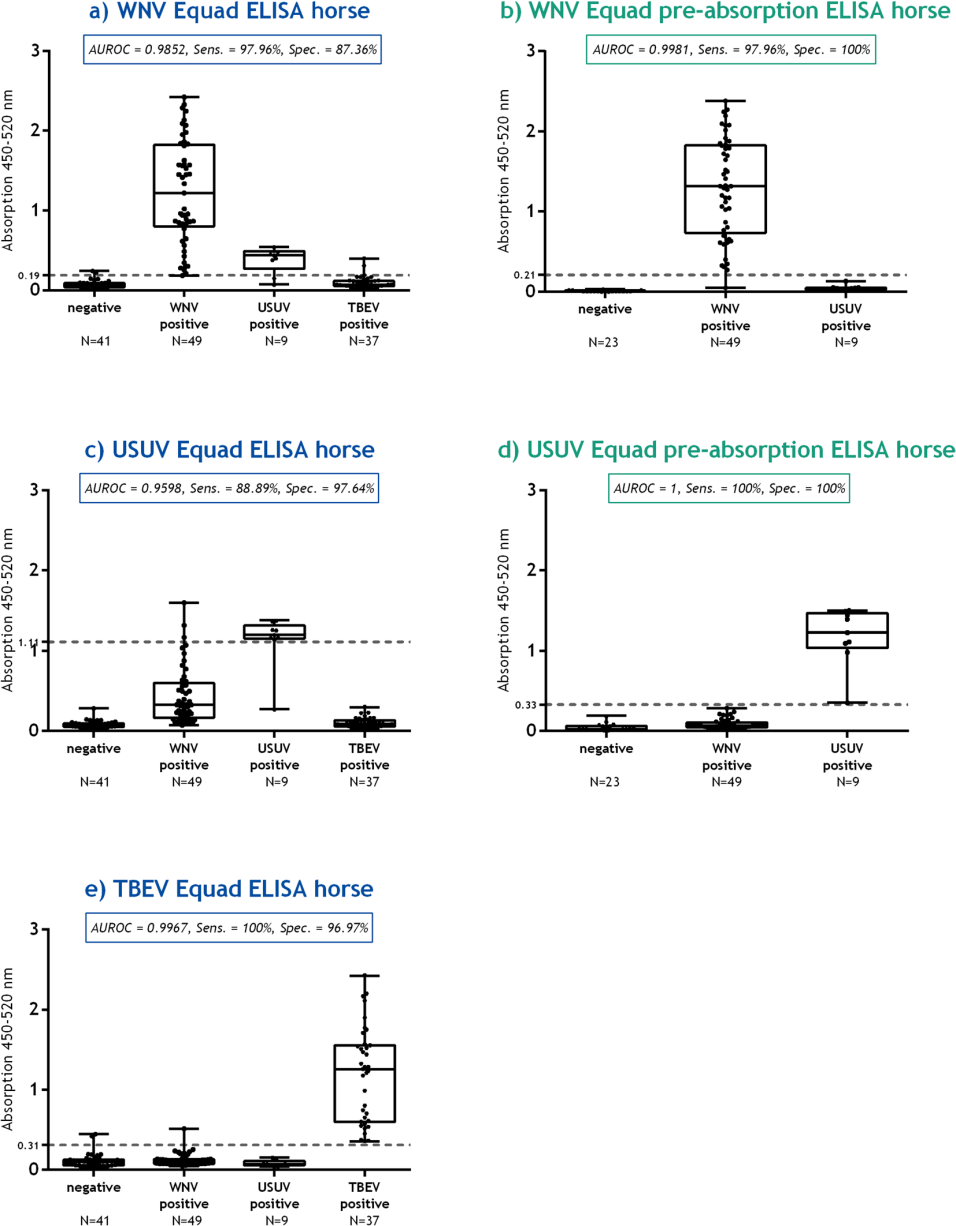
Equad and Equad pre-absorption ELISAs for the differentiation of flavivirus IgG antibodies in horse sera. The sera are categorized according to their antibody status on the x-axis, as verified by virus neutralization tests. The graphs display the area under the curve in receiver-operating characteristic (AUROC) with sensitivity (Sens.) and specificity (Spec.). The calculated cut-off value is indicated by a dashed gray line on the y-axis. (**a)**: WNV Equad ELISA horse sera, 95%CI 0.9715 to 0.9989 (**b)**: WNV Equad pre-absorption ELISA horse sera, 95%CI 0.9934 to 1.003**c)**: USUV Equad ELISA horse sera, 95%CI 0.9091 to 1.010 (**d)**: USUV Equad pre-absorption ELISA horse sera, 95%CI 1.000 to 1.000 (**e)**: TBEV Equad ELISA horse sera, 95%CI 0.9917 to 1.002.

A total of 136 horse sera were analyzed (Tables 1 and 2) using five different ELISA setups (Figs. 3a - e). The corresponding raw data and VNT titers are provided in Supplementary Tables S8 – S11. In the WNV Equad ELISA, a sensitivity of 97.96% and specificity of 87.36% were achieved at a cut-off value of 0.19 (Fig. 3a), resulting in an AUC of 0.9852 in ROC analysis (S8 Fig). In the WNV Equad pre-absorption ELISA (Fig. 3b), the sensitivity remained at 97.96% because of one false negative sample, but the specificity increased to 100% at a cut-off value of 0.21. The AUROC for the WNV Equad pre-absorption ELISA was higher compared to the WNV Equad ELISA with a value of 0.9981 (S9 Fig). Among the nine USUV-positive horse samples, seven sera were false positives in the WNV Equad ELISA (~ 77.78%). In contrast, none of the eight sera were positive in the WNV Equad pre-absorption ELISA, indicating an improvement of the test by including the pre-absorption step. As already described for the WNV Equad ELISA, cross-reactive antibody binding between WNV and USUV positive horse sera was observed for the USUV Equad ELISA (Fig. 3c). The ROC analysis for the USUV Equad ELISA revealed a sensitivity of 88.89% and a specificity of 97.64% at a cut-off value of 1.11, with an AUROC of 0.9598 (S10 Fig). This could subsequently be increased to 1 in the USUV Equad pre-absorption ELISA (Fig. 3d), achieving a sensitivity and specificity of 100% at a considerably lower cut-off value of 0.33 (S11 Fig). The TBEV Equad ELISA (Fig. 3e) for horse sera showed a sensitivity of 100% and a specificity of 96.97% at a cut-off value of 0.31 with an AUC of 0.9967 in the ROC analysis (S12 Fig). Given the minimal cross-reactive binding was observed and the fact that TBEV belongs to a different serogroup than WNV and USUV, it was not considered necessary to perform a pre-absorption ELISA for TBEV.

## Discussion

The increasing prevalence of mosquito-borne flaviviruses, including WNV and USUV, highlights the urgent need for the development of specific serological diagnostics that can differentiate between these viruses. A significant challenge in the serological differentiation of flavivirus infections is the high degree of cross-reactivity between antibodies against closely related viruses. This cross-reactivity leads to a considerable number of false positive results in currently available serological tests, particularly in regions where closely related flaviviruses co-circulate, such as parts of Germany, where WNV, USUV and TBEV are present.

This study focusses on differentiating WNV, USUV and TBEV IgY and IgG antibodies in avian and equine sera. The primary objective was to develop a rapid and reliable ELISA method for distinguishing IgY/IgG antibodies against WNV, USUV and TBEV in susceptible hosts. The antigens used in this study were recombinant envelope proteins with mutations in and near the highly conserved FL epitope^32^. Different Equad ELISA and Equad pre-absorption ELISA protocols were established. Our results demonstrate that these antigens can indeed be used for the specific and sensitive detection of IgY antibodies in duck, goose, chicken and IgG antibodies in horse sera. Equad pre-absorption ELISAs were identified as the optimal method for achieving high performance in differentiating WNV and USUV antibodies.

Equad proteins have been previously used for detecting flaviviruses in human medicine^35,36,44^ and for pilot studies for detection of TBEV and WNV positive equine sera^34,37^. In this study, we extend the applicability of Equad proteins in ELISAs for the differentiation of WNV and TBEV to birds and include USUV as a target flavivirus that co-circulates with WNV and TBEV in many regions in Europe.

Different field studies indicate that unvaccinated horses, zoo birds and non-migratory wild bird species are useful sentinel species for monitoring the circulation of WNV and USUV^45,46^. Previous experimental studies revealed that chickens, ducks and geese are susceptible to WNV and USUV but develop relatively low viral loads in blood, making them less effective as amplifying hosts, with some exceptions noted in geese^47^. Nevertheless, poultry can serve as effective sentinel hosts due to their rapid seroconversion and high antibody titers upon infection with WNV or USUV^39–41^ which has also been described for TBEV in ducks^42^. Hence, serological diagnostics with high specificity and sensitivity are crucial for monitoring WNV, USUV and TBEV in animals. Our data indicate that E proteins containing modified FL domains are well suited to address this need. Using Equad proteins from WNV, USUV and TBEV allowed the accurate differentiation of infections in the vast majority of the animal serum samples investigated. Specific diagnosis of TBEV infections was achieved using the standard ELISA protocol; however, an additional pre-absorption step with the corresponding heterologous antigen was necessary to differentiate between USUV and WNV specific antibodies. The observed higher degree of cross-reactive antibody binding between WNV and USUV as compared to TBEV is expected, as both WNV and USUV belong to the Japanese encephalitis virus serocomplex, whereas TBEV is more distantly related^16,26^.

The negative sera utilized in this study underwent a preliminary screening using the ID Screen^®^ Flavivirus competition ELISA. A recent publication indicates that in competition ELISA tests for flavivirus antibodies detection negative samples can yield positive results for USUV^48^, and this phenomenon has also been observed for TBEV in the past with the same test as used in this study^49^. Consequently, there is a possibility that a small proportion of these sera classified as negative may in fact be positive for USUV or TBEV, thereby exerting a minimal impact on the specificity and sensitivity of the method.

Furthermore, it is noteworthy that horses vaccinated against WNV would also produce positive ELISA results. Therefore, it is crucial to consider unvaccinated horses as sentinels and to ascertain the vaccination status of the tested animals to ensure the accurate interpretation of ELISA results, thereby avoiding the incorrect attribution of a positive ELISA result in a vaccinated horse to a WNV infection.

The current gold-standard for differentiating flavivirus infections on a serological level is the VNT^27^, which has limitations including being time consuming and requiring specialized laboratory infrastructure. Recent advancements in veterinary diagnosis have introduced alternative methods such as protein microarrays and ELISA for horse sera^50,51^. The major advantages of the ELISA format presented in this study are its time-efficiency (1–2 days) and compatibility with standard laboratory settings, making ELISAs a valuable tool for analyzing large sample sets in epidemiological surveillance. It is known that antibodies against WNV, USUV and TBEV can be detected in horses for at least 15 months post infection^52,53^. A minimum persistence of 15 months has also been demonstrated for WNV antibodies in naturally infected wild rock pigeons (*Columba livia*)^54^. Accordingly, antibody monitoring remains feasible long after an infection, further supporting the use of ELISA-based analysis of sentinel animals for monitoring virus circulation. However, a VNT remains necessary whenever precise titers of neutralizing antibodies are required, such as in monitoring individual birds over time, assessing vaccination efficacy in horses or zoo birds^55^, or in the absence of secondary detection antibodies suitable for the variety of bird species^56^. The methods demonstrated herein are applicable to the analyzed species. Nevertheless, further analysis is required regarding the utility of antibodies for other avian species, in light of the divergent detection capacity of the utilized secondary antibody across diverse bird species and families^56^.

The investigation of USUV-positive sera in this study was constrained by the limited availability of positive serum samples from the species examined. Further testing of USUV-positive sera will undoubtedly provide additional insights, particularly from wild birds (e.g. blackbirds), where more USUV-positive sera are available than in the species tested in this study. In addition, reliable data on USUV distribution are needed, as its circulation has not yet been sufficiently documented^57^. Nevertheless, despite the limited number of samples in some cases, clear conclusions can be drawn regarding the differentiation between WNV and USUV using Equad ELISAs and Equad pre-absorption ELISAs. . Additional research on the application of Equad proteins in avian serology, particularly in the context of wild and zoo birds, could advance the current methodology and facilitate a broader diagnostic application.

## Conclusion

In conclusion, we demonstrate that recombinant flaviviral E proteins containing a modified FL domain effectively minimize cross-reactive binding among WNV, USUV and TBEV IgY or IgG antibodies in avian and equine sera when using ELISA methods. The addition of a pre-absorption step with the heterologous antigen significantly reduces cross-reactive binding between the closely related flaviviruses WNV and USUV. The differentiation of WNV, USUV and TBEV in chickens, ducks, geese and horses using Equad proteins is rapid, cost-effective and sustainable. The ability to conduct these ELISAs in standard diagnostic laboratories, combined with the time savings and the reliable differentiation of flavivirus antibodies within the same serocomplex represents significant advancements in the specific and faster serology of flavivirus infections.

## Supplementary Information

Below is the link to the electronic supplementary material.

Supplementary Material 1

Supplementary Material 2

## Data Availability

All data generated or analyzed during this study are included in this published article and its supporting information files.
