# Supplementary material for: Serological differentiation of West Nile, Usutu, and tick-borne encephalitis virus antibodies in birds and horses using mutant E protein ELISAs
Source: Sci Rep. 2025 Aug 6;15:28752. doi: 10.1038/s41598-025-14448-4 (PMC12328586; doi:10.1038/s41598-025-14448-4)
Supplement: Supplementary file 1 — Supplementary Material 1 [file 41598_2025_14448_MOESM1_ESM.pdf]

Serological differentiation of West Nile, Usutu, and tick-borne encephalitis virus antibodies in birds and horses using mutant E protein ELISAs

Anne Schwarzer[1], Ute Ziegler[1], Jasmin Fertey[2], Markus Kreuz[3], Thomas W. Vahlenkamp[4], Martin H. Groschup[1], Sebastian Ulbert[2,*]

Supplementary Figures 1 - 12

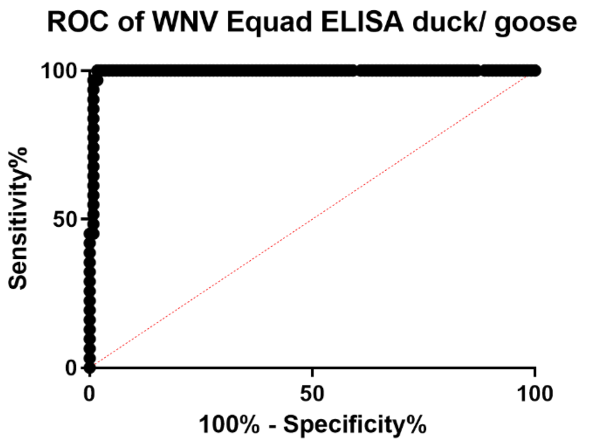

Figure S1: Curve of the receiver operating characteristics analysis of WNV Equad ELISA for duck and goose sera

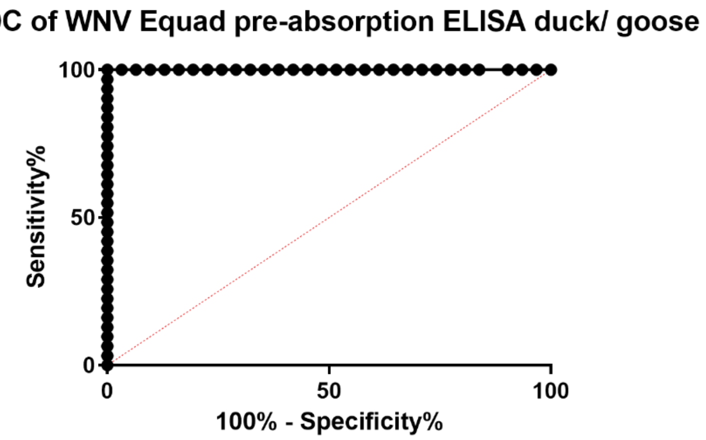

Figure S2: Curve of the receiver operating characteristics analysis of WNV Equad pre-absorption ELISA for duck and goose sera

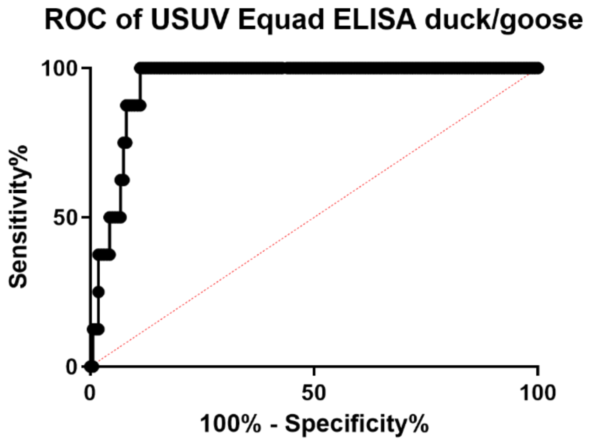

Figure S3: Curve of the receiver operating characteristics analysis of USUV Equad ELISA for duck and goose sera

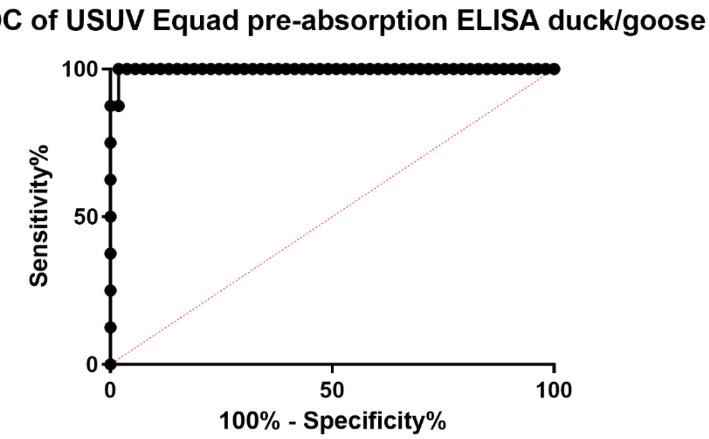

Figure S4: Curve of the receiver operating characteristics analysis of USUV Equad pre-absorption ELISA for duck and goose sera

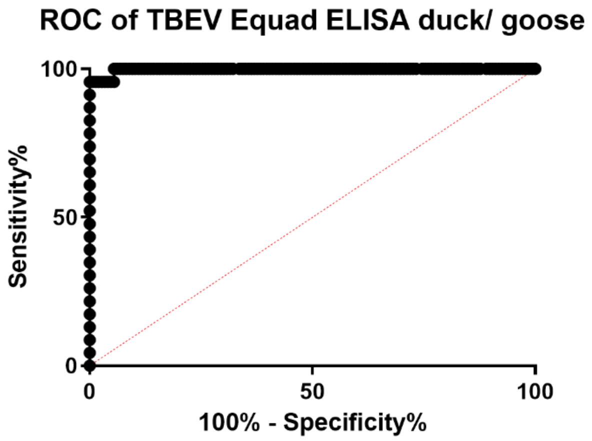

Figure S5: Curve of the receiver operating characteristics analysis of TBEV Equad ELISA for duck and goose sera

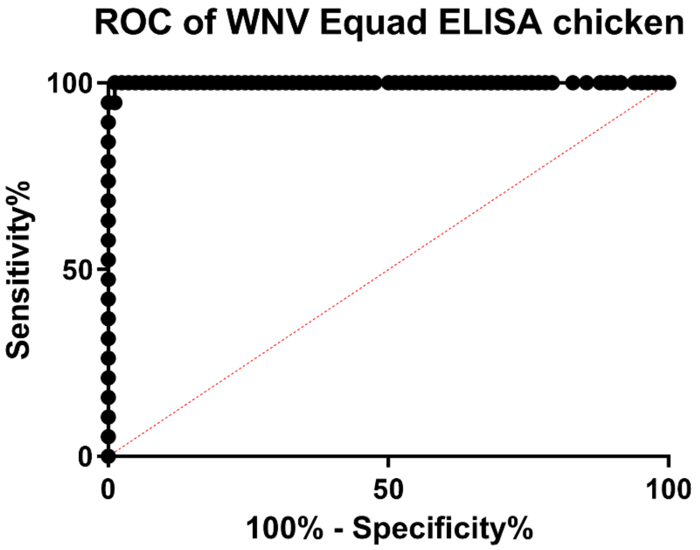

Figure S6: Curve of the receiver operating characteristics analysis of WNV Equad ELISA for chicken sera

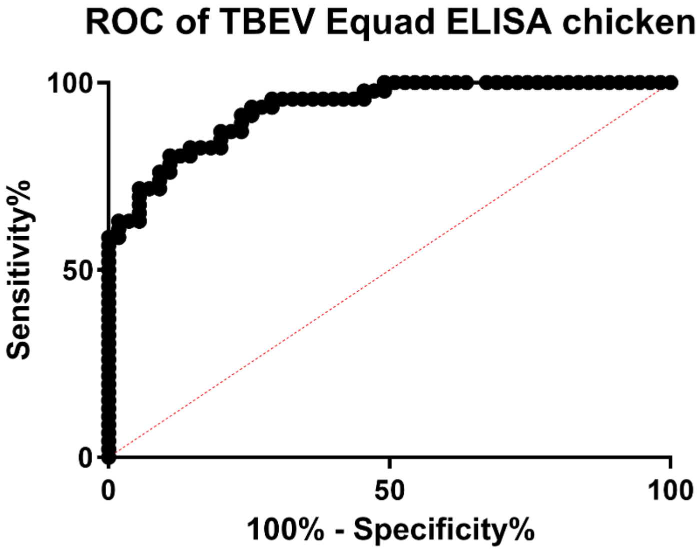

Figure S7: Curve of the receiver operating characteristics analysis of TBEV Equad ELISA for chicken sera

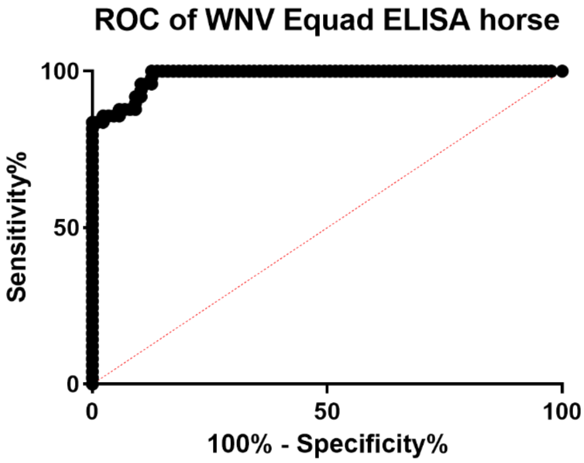

Figure S8: Curve of the receiver operating characteristics analysis of WNV Equad ELISA for horse sera

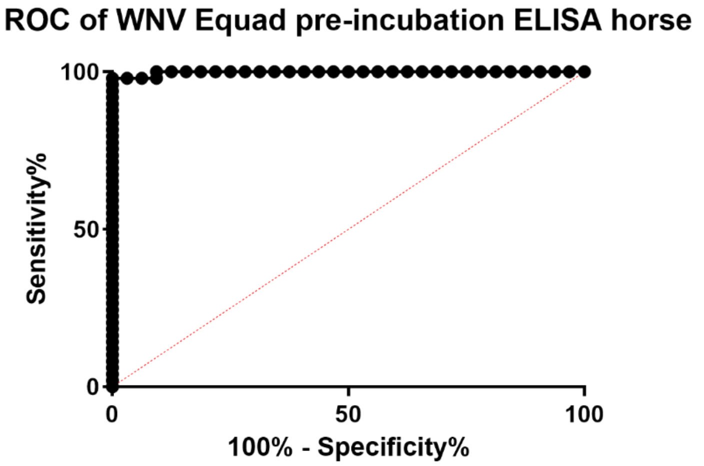

Figure S9: Curve of the receiver operating characteristics analysis of WNV Equad pre-absorption ELISA for horse sera

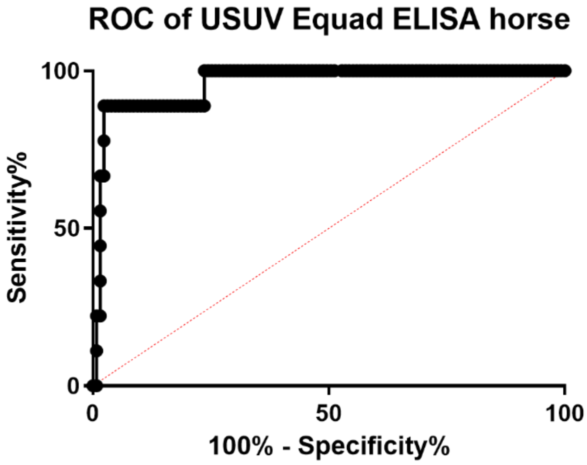

Figure S10: Curve of the receiver operating characteristics analysis of USUV Equad ELISA for horse sera

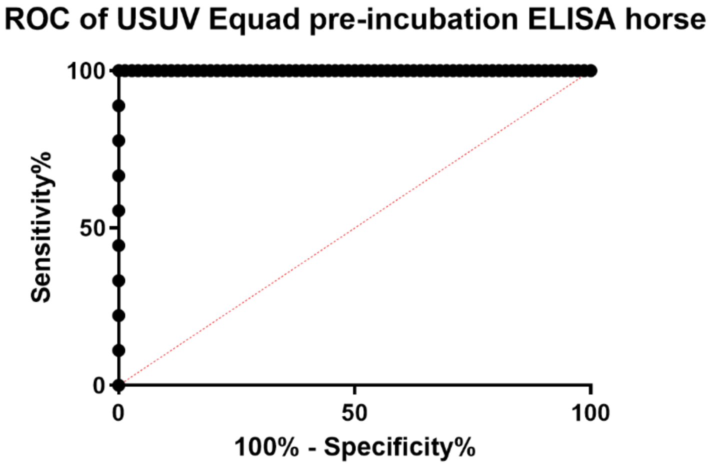

Figure S11: Curve of the receiver operating characteristics analysis of USUV Equad pre-absorption ELISA for horse sera

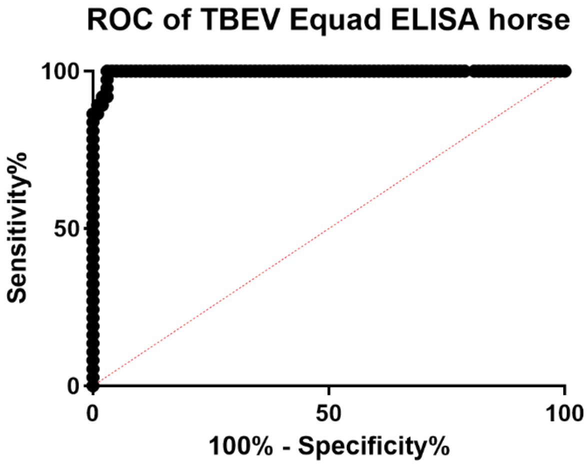

Figure S12: Curve of the receiver operating characteristics analysis of TBEV Equad ELISA for horse sera
